# Assessing the Interactions between Strengths and Risk Factors of Recidivism through the Structured Assessment of Violence Risk in Youth (SAVRY)

**DOI:** 10.3390/ijerph17062112

**Published:** 2020-03-23

**Authors:** Elena Ortega-Campos, Juan García-García, Leticia De la Fuente-Sánchez, Flor Zaldívar-Basurto

**Affiliations:** Health Research Center, Faculty of Psychology, University of Almería, 04120 La Cañada, Almería, Spain; jgarciag@ual.es (J.G.-G.); lfuente@ual.es (L.D.l.F.-S.); flor@ual.es (F.Z.-B.)

**Keywords:** SAVRY, juvenile offenders, recidivism, protective factor, risk factor, risk assessment

## Abstract

Instruments that assess recidivism risk in young people are used widely in the sphere of juvenile justice worldwide. Traditionally, research has focused on the study of risk factors presented by young offenders, and how these relate to criminal recidivism. In present-day research, protective factors have also come into their own, having proven to encourage non-recidivism in young offenders. This paper presents a study carried out with 594 young offenders. The instrument used for assessing risk of recidivism in young offenders was the Structured Assessment of Violence Risk in Youth (SAVRY). In the results found here, one can observe how the young offenders who did not reoffend presented a greater level of protective factors than the repeating offenders. The youths with a prior arrest record scored higher in the risk domains than the reoffenders without a prior arrest record. The case of young repeat offenders who already had an arrest record represents a high-risk profile, or a profile of a criminal career. Crimes committed by young people can be isolated incidents in their life. In most youths, criminal behavior does not persist beyond legal age. Protective factors prove to be important in juvenile justice when planning an individualized intervention for the young offender.

## 1. Introduction

The use of risk assessment tools in juvenile justice continues to rise [1], thanks to their usefulness for professionals who work in identification of youths’ criminogenic needs. Each offender shows certain specific risk and protective factors that must be identified in order to plan an adequate intervention for the individual, in the effort to stem off future offenses [2,3]. Interventions based on the criminogenic needs of young offenders are more effective than general interventions [4].

The prevailing model for explaining criminal behavior in young offenders is the Risk, Need, Responsivity (RNR) model. It identifies youths that require intervention (risk), the criminogenic needs that they present (needs), and the strategies that should be used to maximize the youth’s ability to benefit from the intervention (responsivity) [4,5].

Key to the RNR Model is the assessment of youths’ risk of recidivism based on criminogenic needs, and the risk and protective factors presented by each young offender. Youth who present a greater risk of recidivism should receive a greater number of resources to reduce their probability of reoffending, while those with a low risk of recidivism should not be recipients of large interventions [2].

Risk factors increase the probability of delinquent behaviors [6,7]. Past studies have found a direct relationship between the presence of risk factors in young offenders and an increased probability of recidivism [8,9,10]. Risk factors may be static, that is, they predict criminal behavior but are not modifiable (e.g., a criminal record); or dynamic, that is, modifiable through interventions designed to reduced recidivism. Instruments for assessing risk of recidivism mainly address dynamic risk factors, because of their possibility of change. Static risk factors, despite their nonmodifiable nature, also form part of the risk assessment instruments, because of their importance in predicting juvenile recidivism [11]. The RNR Model is made up of the risk factors which research has identified as being most closely related to delinquent behavior.

Traditionally, protective factors have been defined as characteristics that reduce the likelihood of recidivism [12,13]. The presence of protective factors can compensate for the negative effect of risk factors on the young offender. In today’s research, authors distinguish between protective factors and promotive factors. While protective factors represent the absence of risk factors in a young person (e.g., does not have delinquent friends), promotive factors are characteristics that present a negative relationship to criminal behavior (e.g., has prosocial peers). Protective factors are especially important in assessing risk because of their relation to risk factors; they help reduce recidivism in youths with moderate to high risk [14]. Recent studies have addressed the importance of risk and protective factors in predicting juvenile delinquent careers [15,16,17]. The presence/absence of risk and protective factors in young people leads to different patterns in juvenile delinquent careers. Assessment of risk and protective factors has been established as important in distinguishing nonchronic delinquent careers in adolescents from repeat offenders that persist into adulthood [18]. The identification of specific profiles associated with recidivism provides an opportunity to determine the risk and dynamic criminogenic needs for each young offender, and to provide effective treatment for each young offender [15].

Results indicate a negative relationship between protective factors and recidivism in young offenders, and that protective factors are important in desisting from delinquent recidivism [19]. The specific role played by prediction factors in conjunction with risk factors is a topic of interest for present-day researchers, who focus on the incremental validity of both types [14,20,21].

The use of risk assessment instruments for recidivism is a common practice in juvenile justice [1]. Risk assessment instruments for recidivism help to identify the needs presented by each young offender and help in decision-making about the intervention to be carried out [4]. Two of the most commonly used tools in assessing young offenders’ risk of recidivism are the Structured Assessment of Violence Risk in Youth (SAVRY) [22] and the Youth Service Level/Case Management Inventory (YLS/CMI) [23].

The SAVRY instrument [22] identifies risk and protective factors present in young offenders, with the objective of reducing recidivism through appropriate intervention in protective and risk factors. The SAVRY’s contribution, with respect to other instruments, is its incorporation of the protection factor. Protective factors are predictors of non-recidivism [13] and they help to mitigate the negative effect of risk factors.

The present study has the following objectives: (a) Study the functioning of the SAVRY in a sample of non-English-speaking young offenders; (b) Study whether there were differences in SAVRY scores between the youths who reoffended after their baseline incident (recidivists) and those who did not; and in the recidivists, between those who already had an arrest record prior to the baseline year, and those who did not; (c) Study the predictive capacity of SAVRY scores in recidivists vs. non-recidivist offenders, and in the recidivist youths with and without a prior arrest record; and (d) Study the role of the SAVRY protective factors compared to its risk factors, paying attention to the incremental validity provided by the protective factors.

## 2. Method

### 2.1. Participants

The participants in this study were young people who had at least one open case file with the Juvenile Court of Almería (Spain) for having committed an offense that is penalized under Spanish law. According to the Minor’s Penal Responsibility Act (Organic Law 5/2000), any young person who commits a criminal offense after their 14th birthday, but prior to turning 18, will be judged in juvenile court. For all the youths we selected, a case file had been opened within one particular calendar year (January–December), in the provincial Juvenile Court of Almeria (Spain). The sample was thus representative of the phenomenon of juvenile delinquency in one region of Spain.

The final study sample was composed of 594 young people, primarily (85.4%) boys, with 507 male and 87 female offenders. Regarding nationality, 79% were of Spanish nationality, and the largest non-Spanish nationality was Moroccan (9.6%). The mean age at the time of the offense was 15.63 years and the age range was 14–17; 19.5% were 14 years of age, 25.6% were age 15, 26.9% age 16 and 27.9% age 17. Among crimes committed by young offenders, 42% were crimes against persons and 47% were crimes against property. A judicial measure had been applied to 52.2% of the youths, in response to the offense committed. The most frequently imposed measures were: probation (19.5%) and semi-open detention (6.2%). In regard to schooling, 59.9% of the youths had repeated at least one year in school (Table 1).

### 2.2. Procedure

The data collection process was carried out at the Juvenile Court of Almería (Spain). The information required to complete the SAVRY instrument was collected retrospectively from the case files of the young offenders. These case files include police information regarding the incident reported, the criminal investigation of the facts, the psycho-socio-educational report prepared by juvenile court personnel, and the sentence imposed by the judge. Using the documentation in the young person’s case file, we completed our data collection protocol for this study, which included sociodemographic variables, information relating to young offender’s recidivism, prior criminal records and the SAVRY scores.

Two of the study authors acted as coders. One of the authors coded 100% of the youths’ court records, the second coder coded 30% of the files, selected randomly. Agreement between coders was greater than 95%, with discrepancies solved by consensus. Both coders have a doctorate in Psychology; one has over 20 years’ experience in legal and forensic psychology.

This research study followed the recommendations of the risk assessment guidelines of the Evaluation of Efficacy (RAGEE) Statement [24], and was approved by the University of Almería Ethics Committee (UALBIO2020/017) within the framework of a broader investigation.

### 2.3. Measures

#### 2.3.1. Structured Assessment of Violence Risk in Youth

The SAVRY instrument [22] for risk assessment in young offenders is composed of 4 factors, three of which refer to the young person’s risk—historical (10 items), social (6 items) and individual (8 items)—and one factor represents protective (6 items). The items have closed responses; risk items offer three alternatives (low, moderate and high), and protective items offer two (presence, absence). The SAVRY produces partial scores for each of the factors (historical, social, individual and protective) and one total score. In this study, the Spanish adaptation [25] was used. In order to study reliability of the instrument scores, the Cronbach alpha coefficient was calculated for the partial and total scores, obtaining the following values: α = 0.797, CI 95% (0.772, 0.820) in the historical factor; α = 0.685, CI 95% (0.644, 0.723) in the social factor; α = 0.761, CI 95% (0.730, 0.789) in the individual factor; α = 0.815, CI 95% (0.790, 0.837) in the protective factor; and α = 0.781, CI 95% (0.755, 0.805) for the risk total score [26].

#### 2.3.2. Recidivism and Prior Arrest Records

The measure of recidivism for this study was defined as a new judicial case being opened against the young offender by the prosecuting authority. The judicial case could have been opened because of a complaint, or directly by the public prosecuting authority. The study period for criminal recidivism were the two years following the baseline calendar year for all youths [27,28,29,30]. The prior criminal record was measured by reference to the presence of a judicial case in the youth’s judicial record. The study period was the two years prior to the case committed during the reference year.

### 2.4. Data Analysis

Cronbach’s alpha reliability coefficient was calculated in order to study the internal consistency of partial and total SAVRY scores. In evaluating the reliability coefficients, we followed the recommendations of George and Mallery [31].

Proper functioning of the SAVRY was verified using several approaches. First, instrument scores were descriptively analyzed by calculating the mean scores, standard deviations, minimum and maximum values and the Spearman-Brown correlation coefficients. We performed non-parametric tests of differences of means (Mann-Whitney *U*) between the SAVRY scores. The contrast statistic was accompanied by estimating the effect size [32,33] and the Bayes factor, taking the values proposed by Jeffreys [34] as our reference.

To quantify the prediction strength of the SAVRY for predicting recidivism, AUCs (area under the curve) were calculated for the instrument scores. To interpret these AUCs, we took the following reference: AUCs between 0.55 and 0.63 show low predictive ability, between 0.64 and 0.70 indicate medium predictive ability, and higher than 0.71 indicate good predictive ability, complemented by estimation of the effect size using Cohen’s *d* index [35].

In complementary fashion, in order to study how well the SAVRY domains predict young offenders’ recidivism, logistic regression models were calculated for each of the groups. Model interpretation was based on the statistical significance associated with each domain, the percentage that was correctly predicted, and the value of Nagelkerke’s *R*^2^ coefficient. Our interpretation of Nagelkerke’s *R*^2^ followed the recommendations of Cohen [32]. For each domain entered in the logistic regression, we calculated the value of *Exp(b)* in Odds Ratio (OR) format and its 95% confidence interval. OR higher than 1 indicates greater likelihood of occurrence; OR less than 1 indicates lesser likelihood of occurrence [36]. For the comparison of the best competitive model, we calculated the difference of the deviances and their statistical significance, and the Bayes factor was estimated through the BIC difference (Bayesian Information Criterion). Statistical analyses were carried out using SPSS version 25 (IBM Corp., Armonk, NY, USA) and JASP version 0.9.2 (University of Amsterdam, Amsterdam, The Netherlands).

## 3. Results

Descriptive statistics of the two groups of young offenders in this study were calculated. The first group was formed of the entire set of young offenders for whom a judicial case was opened during the baseline calendar year, and the second group was the subset of young offenders who reoffended in the two years following.

The first group obtained mean values of 6.71 with a standard deviation of 7.84 on the total SAVRY score. In the SAVRY domains, the highest mean score was obtained in the historical domain 3.51 (SD = 3.27) and the lowest score in the social domain 2.12 (SD = 2.37); mean scores in the individual and protective domains were similar, 2.97 and 2.99, respectively. Regarding correlations, values fell between 0.67 and 0.89. The historical domain produced values of close to 0.7 for correlations with the remaining domains, and 0.82 with the total SAVRY score. Correlation values of the social domain with the individual domain and the protective domain were above 0.7, and 0.87 with the total score. The protective domain showed the highest correlation with the individual domain (−0.79). Correlations between the total score and the SAVRY domains are above 0.82.

The group of young offenders who reoffended presented mean values of 10.57(8.26) on the total SAVRY score. In the domains, the highest mean score was found in the historical domain (4.84), followed by the individual domain (4.39), the social domain (3.14) and the lowest score in the protective domain (2.11). Correlation coefficients have adequate magnitude, their calculated values falling between 0.56 and 0.71. The protective domain showed coefficients of −0.56 with the historical domain, −0.71 with the social domain, −0.68 with the individual domain, and −0.82 with the total SAVRY. The total SAVRY score presents coefficients higher than 0.83 with all domains except the protective domain (r = −0.82).

Table 2 presents calculations of the mean scores and standard deviations, as well as the contrast of nonparametric means, effect size estimates for each between-group comparison, and Bayes factor estimates. Comparisons were made between the two groups mentioned above. First, we compared the reoffenders to the non-reoffenders. The reoffenders presented significantly higher scores from a statistical viewpoint in all the risk scores and in the total SAVRY score, in comparison to the young offenders who did not reoffend. In contrast, the non-reoffenders group presented significantly higher scores than the reoffenders group in the protective domain. Effect size estimates for the comparisons were between 0.73–0.90, representing large effects. The Bayes factor was calculated for all the comparisons, obtaining scores over 100.

Following the study of recidivism after the baseline year, our study focused on reoffenders and the possible role of a prior arrest record (before the baseline year) on their SAVRY scores. The group of reoffenders without a prior arrest record contained 138 youths, while there were 73 reoffenders who already had a record before the baseline incident. As seen in Table 2, statistically significant differences are found in the total score and in the risk domains of the SAVRY, where higher mean scores are presented by the group of youths with a prior arrest record. Regarding the protective factor, no statistically significant differences were found between the two groups; youths without a prior arrest record presented a mean score of 2.24 (SD = 1.61), while the mean score for the youths with an arrest record was 1.86 (SD = 1.61). Effect size estimates fell between 0.24–0.44. The Bayes factor estimate was over 100 in the historical domain, and over 30 in the individual domain and in the SAVRY total.

AUCs were calculated for each total score and for the risk and protective domains of the SAVRY, for the whole set of offenders and for the reoffenders. For the set of all young offenders, the AUCs were statistically significant in predicting recidivism, showing values higher than 0.7 for both the total score and for the risk and protective factors. When the AUCs were calculated for the group of reoffenders only, predictive capacity was statistically significant for the SAVRY total score and for the risk domains. The historical factor presented an AUC greater than 0.70, while the total score and the social and individual factors presented scores between 0.62 and 0.69. In the protective domain, the AUC calculation was not statistically significant (Table 3).

Table 4 presents the logistic regressions for the whole set of young offenders and for the reoffender subset, the first step includes the total SAVRY score and the second step adds the protective factor. For the whole set of young offenders, both the SAVRY global score and the protective domain are statistically significant. Higher scores on the SAVRY indicated greater likelihood that the young people would commit another offense (OR > 1), while the protective factor was related to a lower likelihood of recidivism in the young people (OR < 1). In the set of all offenders, the risk score significantly predicted future recidivism (OR = 1.066), while the protective factor was significantly related to non-recidivism (OR = 0.812). In the recidivist group, both the risk score (OR = 1.168) and the protective domain (OR = 1.594) were statistically significant. In this case, whether for the whole sample of offenders or for the recidivists only, introduction of the protective domain leads to improved goodness-of-fit to the model, and more markedly so in the sample of recidivists.

The data in Table 5 are presented in order to study the direct and mediating effects of the SAVRY risk and protective factors in recidivism of the young offenders. For the total group of young offenders, we present a model where the protective factor has a direct effect on the young people’s recidivism; the model is predictive from a statistical point of view and has an inverse relation to recidivism (OR = 0.653). In the mediating model, we present a first block with the protective factor and the SAVRY score, corresponding to Table 4. The protective factor, the risk factor and the interaction between the two is studied in a second block, with results showing a statistically significant interaction between the factors (*p* < 0.001).

For the set of reoffenders, in the direct model, the protective factor does not have a statistically significant relation to whether the youths had a previous arrest record. By contrast, in the mediated model, both factors are statistically significant in the first step, with OR higher than one for both the protective factor and the total SAVRY score. In the second step of the logistic regression analysis, the interaction between factors was not statistically significant, unlike the risk and protective domains. In comparing the proposed models, there is a statistically significant improvement in the whole sample of offenders, with respect to the prior models, when the interaction is included. In the case of the recidivists only, this statistically significant improvement of the model does not occur.

In order to gain a better understanding of the protective factor, with more connection to its use in professional practice, the study sample was divided into three groups according to the young offenders’ protective factor scores. The 25th and 75th percentiles were calculated, thereby dividing the sample into three groups: youths who showed a deficit in the protective factor (<P_25_), youths with average scores in the protective factor (P_25_–P_75_) and youths with high scores in the protective factor (>P_75_) [20]. The group with average scores in the protective factor was compared to the group with deficient scores and the group with higher scores (Table 6). In the risk group (youths with low scores vs. youths with average scores), both recidivism and existence of a prior arrest record in the recidivists (level of recidivism) showed statistically significant scores, in both cases with ORs below one. Youths with a deficit in the protective domain presented higher scores in recidivism and in level of recidivism. In the protective group (youths with high scores vs. youths with average scores), we find statistically significant inverse relations with recidivism, that is, youths with high scores in the protective domain present lower risk of recidivism. No statistically significant relations were found in the sample of recidivists with and without an arrest record.

## 4. Discussion

The objective of this study was to verify the functioning of the SAVRY in a sample of non-English-speaking young offenders. The results indicate that the SAVRY adequately distinguishes between recidivist vs. non-recidivist young offenders. The young offenders presented significantly higher scores in the risk domains of the instrument, while the non-recidivists presented a higher score in the protective domain [37]. These results concur with those obtained in different studies with both English-speaking and non-English-speaking young offenders. In this way, the SAVRY has demonstrated adequate functioning and discrimination between recidivist vs. non-recidivist young offenders, regardless of the nationality of the study [29,37,38].

The present study describes results with regard to the scores obtained by repeating offenders with and without an arrest record prior to the baseline year. The youths with a prior arrest record scored higher in the risk domains than the reoffenders without a prior arrest record. The case of young repeat offenders who already had an arrest record represents a high-risk profile, or a profile of a criminal career [39]. Crimes committed by young people can be isolated incidents in their life, in this case, during adolescence. In most youths, criminal behavior does not persist beyond legal age. However, there is a small group of youths who repeat criminal behavior over most of their lifetime, both as adolescents and as adults [40]. In the case of the reoffenders, the only domain in which they do not present statistically significant differences is the protective domain. The fact that there is no distinction in the protective factor between recidivists with and without previous convictions, despite their difference in criminal profile, may be due to the fact that youths with an arrest record have already gone through the juvenile justice system; they have been the beneficiaries of intervention programs that work on protective factors, even though these programs failed to prevent criminal recidivism, due to other aspects of the youth’s profile. Thus, just as recidivism patterns in high-risk young offenders are not affected by the number of risk factors that are present [13], they may likewise be unaffected by protective factors of the protective type. The factors assessed in the SAVRY can be established as protective and not promotive in nature, leading us to consider a need for further research into the latter. Finally, the low incidence of underage recidivists with a high score in protective factors makes it difficult to generalize appropriately.

Not all protective and dynamic risk factors have the same capacity to influence young offenders. Influence capacity and type of relationship must be taken into account when planning interventions, in order to be effective for each young offender [41]. Only a minor part of the identified criminogenic needs are addressed in interventions with juvenile offenders [42]. The identification of profiles associated with recidivism allows for the determination of the risk and dynamic criminogenic needs for each young offender and the planning of personalized treatment [15].

Risk assessment instruments for young offenders do not study the causes of juvenile delinquency, each youth has personal reasons that lead him or her to commit an offense. Risk assessment instruments identify the criminogenic needs of each young offender, which are the specific needs that should be worked on. Intervention programs should pay attention to the risk factors that youths present, and to characteristics that may help the program to work better. The score obtained by a young offender on any risk assessment instrument is only the starting point from which professionals who work in youth reinsertion are to design the intervention that is best suited to the youth’s needs [15,43].

Among the evidence contributed here, we offer a study of the SAVRY’s predictive ability through AUC calculations. These values are similar to those found in studies from different countries, using both English-speaking and non-English-speaking samples [27,38,44,45], and are similar to meta-analysis data of risk assessment instruments in young offenders [46]. Our results uphold the utility and good functioning of the SAVRY in contexts and languages outside of North America, making it feasible to compare studies regardless of the youths’ nationalities.

The presence of a greater number of protective factors in the group of non-recidivist young offenders supports the thesis of the importance of such factors in reducing or preventing criminal recidivism in juvenile justice [13,26,47]. Protective factors are predictive and they play an active role in preventing juvenile recidivism [19,37,48]. Given the fundamental role of protective factors in reducing criminal behavior, it is essential that the different instruments for assessing risk of recidivism include the protective factors presented by the young offenders. An intervention for reducing delinquency should be planned from the basis of a previous study of the youths’ protective factors. The SAVRY includes “Low interest/commitment to school or work” as a risk factor, and also includes “Strong commitment to school or work” as a protective factor. The instruments should include not only protective factors, understood as the absence of a risk factor, but should also take into account the importance of promotive factors. Such variables, while they do not represent the absence of a risk factor, increase the likelihood of the young person not reoffending. Results from a recent meta-analytical study indicate that risk assessment tools remain unchanged, and they support the need to significantly improve risk assessment tools in mental health and justice, including risk factors according to their demonstrated importance in reducing criminal behavior [49].

Regarding incremental validity of the protective factors, this study found evidence to support this incremental validity, with differential effects according to the sample of all offenders or of recidivists only. This result reinforces the importance of protective factors in reducing and preventing juvenile criminal behavior, so it was to be expected that protective factors would add incremental validity to the risk factors in the assessment instruments. Nevertheless, there are studies that do not find statistically significant results of increased incremental validity from protective factors [14,20,50], even though the same studies underscore their importance as principal factors in implementation of youth interventions [14]. Based on our study results, however, one must clearly differentiate the types of protective factors and the reoffender’s type of profile. Bocaccini [51] emphasizes the need to continuously incorporate evidence of validity, since the assessment of recidivism risk is dynamic in nature and is influenced by cultural differences [52], a reality that is clearly reflected in our study.

## 5. Future Lines Research

The role of protective factors in young offenders needs further research. Many of the risk assessment instruments for young offenders do not include protective factors. The assessment is made based only on the risks they present, without taking into account the protective factors. The recent distinction between protective and promotive factors needs to be taken further, both in studies to identify which are the protective and the promotive factors in young offenders, and in the inclusion of these types of factors in risk assessment instruments. The scoring of protective factors should be done with caution, as a score of zero on an instrument’s protective factor does not mean that the young offender has no strengths [20]. Protective and promotive factors should be studied in non-recurrent and non-offending young people with the aim of improving interventions for the prevention of offending behavior.

## 6. Conclusions

In the study of recidivism in young offenders, efforts were traditionally dedicated to identifying the risk factors presented by young people. Current research has advanced approaches to young offenders and now focuses on studying what protective factors young people who do not reoffend have.

This work has shown how young non-recidivist offenders have higher levels of protective factors. In order to prevent crime among young people, it is essential to know which are both protective and promotional factors that help to discourage delinquent behaviour among young people.

## Figures and Tables

**Table 1:** Frequency and percentage of the juvenile’s variables.

Variables	% (*n*)
Age	
14 years	19.5% (116)
15 years	25.6% (152)
16 years	26.9% (160)
17 years	27.9% (166)
Gender	
Male	84.5% (507)
Female	14.6% (87)
Criminal measures	
Probation	19.5% (116)
Semi-open detention	6.2% (37)
Criminal behaviors	
Crimes against persons	42% (250)
Crimes against property	47% (279)

**Table 2:** Descriptive statistics, nonparametric contrasts of means (*U*) and estimated effect size.

SAVRY	M (SD)	M (SD)	Z (p-BF_10_)	Cohen’s d
Total Sample	Does not reoffend *n* = 383	Reoffends *n* = 211		
SAVRY_Historical_	2.77 (2.87)	4.84 (3.51)	−8.682 *^,a^	0.7625
SAVRY_Social_	1.55 (2.09)	3.14 (2.50)	−8.407 *^,a^	0.735
SAVRY_Individual_	2.18 (2.40)	4.39 (2.71)	−10.081 *^,a^	0.9086
SAVRY_Protective_	3.47 (1.84)	2.11 (1.62)	−8.531 *^,a^	0.7473
SAVRY_Total_	4.56 (4.56)	10.57 (8.25)	−9.763 *^,a^	0.8744
Recidivists	No prior arrest record *n* = 138	With prior arrest record *n* = 73		
SAVRY_Historical_	4.03 (3.16)	6.38 (3.64)	−5.282 *^,a^	0.444
SAVRY_Social_	2.77 (2.38)	3.84 (2.60)	−3.028 * (4.3)	0.2504
SAVRY_Individual_	3.77 (2.36)	5.57 (2.95)	−4.367 *^,b^	0.3643
SAVRY_Protective_	2.24 (1.61)	1.86 (1.61)	−1.783 (0.075; 0.47)	0.247
SAVRY_RTS_	8.69 (7.40)	14.14 (8.63)	−4.529 *^,b^	0.3782

* = *p* <.001; ^a^ = BF_10_ > 100; ^b^ = BF_10_ > 30.

**Table 3:** AUC (area under the curve) analysis, confidence interval 95% and effect size.

SAVRY	AUC	CI 95%	Standard Error	*p*	d
Total Sample					
SAVRY_Historical_	0.711	(0.669, 0.753)	0.021	<0.001	0.786
SAVRY_Social_	0.702	(0.658, 0.745)	0.022	<0.001	0.749
SAVRY_Individual_	0.747	(0.706, 0.788)	0.021	<0.001	0.665
SAVRY_Protective_	0.713	(0.674, 0.749)	0.022	<0.001	0.795
SAVRY_Total_	0.737	(0.695, 0.779)	0.021	<0.001	0.896
Recidivists					
SAVRY_Historical_	0.718	(0.647, 0.790)	0.036	<0.001	0.815
SAVRY_Social_	0.626	(0.706, 0.788)	0.041	0.003	0.321
SAVRY_Individual_	0.681	(0.605, 0.758)	0.039	<0.001	0.665
SAVRY_Protective_	0.427	(0.346, 0.508)	0.041	0.081	0.260
SAVRY_Total_	0.689	(0.613, 0.765)	0.039	<0.001	0.697

**Table 4:** Incremental validity of protective factors (logistic regression).

SAVRY	Recidivist			Recidivist Level		
	B (SE)	Exp (b) (CI 95%)	Z (*p*)	B (SE)	Exp (b) (CI 95%)	Z (*p*)
Block 1						
SAVRY Total	0.103 (0.014)	1.108 (1.078, 1.139)	7.318 (<0.001)	0.084(0.02)	1.087 (1.046, 1.130)	4.235 (<0.001)
		R^2^ = 0.174			R^2^ = 0.131	
Block 2						
SAVRY Total	0.064 (0.021)	1.066(1.024, 1.110)	3.086 (0.002)	0.156 (0.034)	1.168 (1.092, 1.250)	4.535 (<0.001)
Protective factors	−0.208 (0.080)	0.812(0.695, 0.949)	−2.620 (0.009)	0.466 (0.160)	1.594 (1.166, 2.180)	2.921 (0.003)
	ΔΧ^2^ (1) = 6.518 *p* = 0.01 ΔBIC = 0.13 R^2^ = 0.131			ΔΧ^2^ (1) = 9.354 *p* = 0.002 ΔBIC = 4 R^2^ = 0.185		

*Note.* Recidivist ‘Yes’ coded as class 1. Recidivist Level ‘arrest record’ coded as class 1. ΔΧ^2^ = Deviance Model 2- Deviance Model 1. ΔBIC = BIC Model 2- BIC Model 1.

**Table 5:** Direct vs. buffering effect model (logistic regression).

SAVRY	Recidivist			Recidivist Level		
	b (SE)	Exp (b) (CI 95%)	Z (*p*)	b (SE)	Exp (b) (CI 95%)	Z (*p*)
Direct effect model						
Protective factor	−0.426 (0.052)	0.653 (0.589, 0.723)	−8.175 (<0.001)	−0.149 (0.097)	0.861 (0.712, 1.042)	−1.540 (0.124)
			R^2^ = 0.163			R^2^ = 0.017
Buffering model						
Block 1						
Protective Total	−0.208 (0.080)	0.812 (0.695, 0.949)	−2.620 (0.009)	0.156 (0.034)	1.168 (1.092, 1.250)	4.535 (<0.001)
Risk Total	0.064 (0.021)	1.066 (1.024, 1.110)	3.086 (0.002)	0.466 (0.160)	1.594 (1.166, 2.180)	2.921 (0.003)
			R^2^ = 0.131			R^2^ = 0.185
Block 2						
Protective Total	−0.281 (0.083)	0.755 (0.641, 0.889)	−3.368 (<0.001)	0.377 (0.183)	1.459 (1.019, 2.088)	2.061 (0.039)
Risk Total	0.036 (0.020)	1.037 (0.997, 1.078)	1.846 (0.067)	0.138 (0.036)	1.148 (1.070, 1.231)	3.869 (<0.001)
Risk x Protective	0.034 (0.009)	1.035 (1.016, 1.054)	3.648 (<0.001)	0.020 (0.015)	1.020 (0.990, 1.051)	1.306 0(.192)
	ΔΧ^2^ (1) = 14.665 *p* ≤ 0.001 ΔBIC =8.279		R^2^ = 0.216	ΔΧ^2^ (1) = 2.134 p = 0.144 ΔBIC =3.217		R^2^ = 0.197

Recidivist ‘Yes’ coded as class 1. Recidivist Level ‘arrest record’ coded as class 1. ΔBIC = BICModel2- BICModel1.

**Table 6:** Effects of low and high Structured Assessment of Violence Risk in Youth (SAVRY) protective total scores.

	Risk Effect (Low Scores on SAVRY Protective Factors) vs. Average Scores				Protective Effect (High Scores on SAVRY Protective Factors) vs. Average Scores			
	B (SE)	Exp (b) (95% CI)	Z (*p*)	r^2^	B (SE)	Exp (b) (95%CI)	Z (*p*)	r^2^
Recidivism	**−0.995 (0.203)**	0.370 (0.248, 0.551)	−4.893 (<0.001)	0.066	−1.801 (0.477)	0.165 (0.065, 0.421)	−3.775 (<0.001)	0.083
Recidivism level	−0.680 (0.301)	0.506 (0.281, 0.914)	−2.258 (0.024)	0.033	1.344 (0.934)	3.833 (0.615, 23.902)	1.439 (0.150)	0.057

*Note.* Recidivist ‘Yes’ coded as class 1. Recidivist Level ‘arrest record’ coded as class 1. Risk effect “low scores” coded as class 0. Protective effect “high scores” coded as class 1.
